# Time between First and Second Posttonsillectomy Bleeds

**DOI:** 10.1155/2017/3275683

**Published:** 2017-08-07

**Authors:** Sheriza Hussain, Ashley P. O'Connell Ferster, Michele M. Carr

**Affiliations:** ^1^Department of Anesthesiology, Georgetown University, Washington, DC, USA; ^2^Division of Otolaryngology-Head & Neck Surgery, Department of Surgery, The Pennsylvania State University College of Medicine, Hershey, PA 17033, USA; ^3^Department of Otolaryngology-Head and Neck Surgery, West Virginia University, Morgantown, WV 26505, USA

## Abstract

**Objective:**

To determine the time between first and recurrent posttonsillectomy hemorrhages (PTHs) and find factors related to multiple PTHs.

**Methods:**

Retrospective chart review.

**Results:**

Of 112 patients, 91 had one PTH, while 21 had recurrent PTHs. Patients with recurrent bleeds had significant differences (*P* < 0.05) in indication for tonsillectomy (47.6% had recurrent tonsillitis), prior cardiac conditions (28.6%), transfusions (9.5%), and hematology consults during the initial PTH visit (19%). Bleeding occurred at a mean of 6.1 (range 1–13) days for the first episode and 10 (range 9–18) days for the second episode as compared to 6.65 (range 1–18) days for those who bled once. Recurrent PTH patients were less likely to have had surgical control of the initial bleed (*P* < 0.05). Patients who bled at 7 days or later were more likely to bleed again within one day (OR 23.0, RR 12). Regression analysis showed that age, failure to have operative control of PTH, and surgical indication were most important in predicting recurrent PTH.

**Conclusions:**

Operative control of PTH is associated with a better outcome than monitoring alone. Patients with PTH within 7 days of tonsillectomy are likely safe to discharge soon after treatment; those who bleed after 7 days should be monitored longer.

## 1. Introduction

Posttonsillectomy hemorrhage, or PTH, is a serious complication of tonsillectomy and, in severe cases, may lead to death [1, 2]. It occurs at a rate of approximately 3.5%, with 0.9% of patients requiring surgical intervention and 0.04% requiring transfusion [2, 3]. Past studies have reported a higher incidence of PTH in adult male patients [4]. According to the postoperative time elapsed, PTH can be classified as either primary, which occurs within the first 24 hours of surgery, or secondary, which occurs after the first 24 hours, typically between days 5 and 10 [2]. Causes of either are not well defined.

Hospital readmission and revisit rates after surgical procedures have increasingly been used as a means of evaluating quality of care. Hospital revisits due to surgical complications result in additional morbidity for patients, as well as extra costs. With the changing face of healthcare, providers and hospitals may be penalized for having a higher than average number of hospital readmissions [5].

The question that prompted this study was the following: do patients with PTH need to be admitted overnight and can we predict who will bleed a second time? Our goal was to identify factors related to multiple PTHs in individual patients including time between bleeds in order to improve management of these patients.

## 2. Materials and Methods

This study was conducted through a retrospective chart review and was approved by our Institutional Review Board (#307). All patients, both pediatric and adult, who presented to Penn State Hershey Medical Center (PSHMC) for PTH from January 2003 to July 2014, were identified using Current Procedural Terminology (CPT) codes 42960, 42961, and 42962 and reviewed for the study. At this institution, tonsillectomy is typically an outpatient procedure. Tonsillotomy patients were excluded. Patients who bled following tonsillectomy prior to leaving the hospital (primary hemorrhages) were excluded from this study. This study included patients who had their surgery at other centers but presented to our facility with post-op bleeding, and it does not include patients who had tonsillectomy at our hospital but presented elsewhere with post-op bleeding.

A total of 132 patients were initially identified as having had posttonsillectomy hemorrhage. Twenty were excluded because they were primary bleeds, leaving a total of 112 patients in the study. The patients were then divided into two groups: Group A, which included patients with one posttonsillectomy bleed (*N* = 91), and Group B, which included patients with two or more posttonsillectomy bleeding episodes (*N* = 21). Within Group B, 20 patients bled twice following tonsillectomy, and 1 patient bled three times.

Data collected for each patient included demographics, indication for surgery, number of days between events (tonsillectomy and bleeds), method of tonsillectomy and for control of bleeding, transfusions, bloodwork, and consults obtained. Statistical analysis was performed using the Statistical Package for the Social Sciences (SPSS 22) software program. The chi-square test, *t*-test for equality of means, and likelihood ratios were used to determine statistical significance, which was set at *P* < 0.05.

## 3. Results

In this group, 17.2% of patients with a posttonsillectomy hemorrhage bled again after medical management—either monitoring with supportive therapy or a procedure to control the bleeding was commenced. At the time of tonsillectomy and PTH, the average age of patients in Group A was 16.7 years, while the average age of Group B was 22.3 years. Patients in Group B were more likely to be male, have a higher BMI, and be Caucasian, but these differences were not statistically significant (Table 1). The most common indication for tonsillectomy for Group A was obstructive sleep apnea, or OSA, which represented 46.7% of patients in Group A. Other indications were recurrent tonsillitis (36.7%), combined OSA and recurrent tonsillitis (13.3%), and malignancy (1.1%). Indications for tonsillectomy in Group B are seen in Table 2. There was a significant difference (*P* < 0.05) for indication for tonsillectomy, with more patients in Group A having obstructive sleep apnea as their indication (47.3% versus 27.3% in Group B) and more patients in Group B having recurrent infection as their indication (47.6% versus 36.3% Group A). Timeframe of bleed based on indication for surgery is also seen in Table 3.

Method of tonsillectomy was not significantly different between Groups A and B. Cautery tonsillectomy was done in 84.8% of the total group and coblation tonsillectomy in 4.5%. The method was unknown in 10.7% because the surgery had been done at an outside institution and records were not transferred.

Bloodwork results were not significantly different between the groups. Just over half of the patients had bloodwork performed, based on the clinical scenario rather than a protocol. Twenty-five percent of INR results were elevated (range was 1 to 1.31, with normal range 0.9–1.1). Forty percent of PT results were elevated, range was 12.10 to 15.40 (normal range 12.0 to 14.2 seconds), and all PTT results were normal (range 23 to 31, normal range 23–35 seconds). Of the patients who had a hemoglobin level determined, 82.1% were below the institutional normal range. Hematocrit was below the normal range in 93.6% of those who had the test performed. Thirty-two percent were above the normal range for white blood cell count. There were no coagulopathies identified in our group. Transfusion was done for 1 (1.1%) Group A patient and 2 (9.5%) Group B patients. A Hematology consult was obtained for 4 (4.4%) Group A patients and 4 (19.0%) Group B patients.

The mean number of days post-op where bleeding occurred for recurrent bleed patients was 6.1 (range 1 to 13) days for the first episode and 10 (range 9 to 18) days for the second episode. Five patients (3 children and 2 adults) bled again within approximately one day of their first admission (3 within 24 hours), and the rest bled later than 7 days post-op. Of these, only 1 (a child) had operative control of the first bleed. Half of all patients who bled on Day 7 or later post-op bled again within a day. The odds ratio for bleeding within 1 day if the first bleed occurred at 7 days or later was 23.0 (95% CI 1.07–494.60) and relative risk was 12.0 (95% CI 0.75–192.87). Details appear in Table 3 and Figure 2.

**Figure 1:**
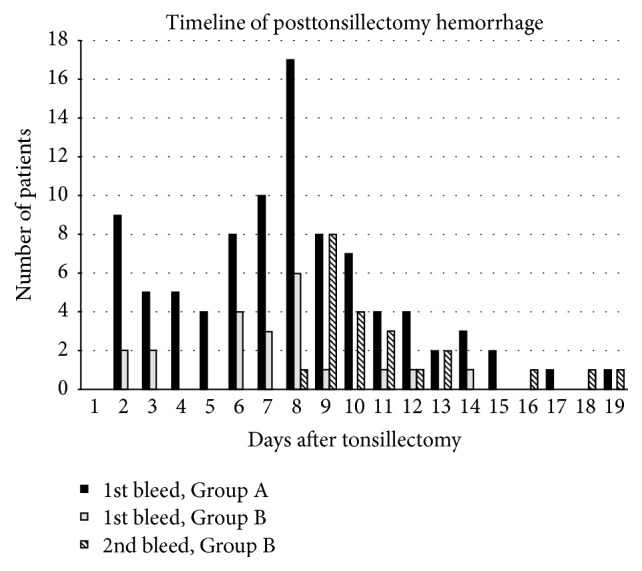
Summary of number of patients who bled on each day after tonsillectomy.

**Figure 2:**
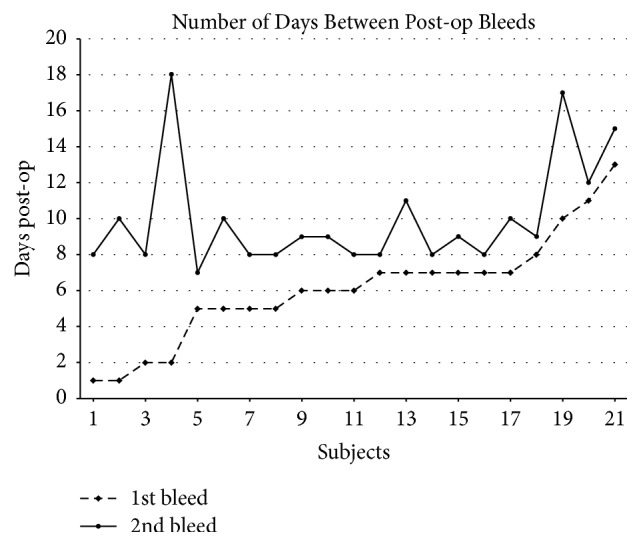
Number of days between posttonsillectomy bleeds.

The mean number of days post-op where bleeding occurred for the patients that bled only once was 6.65 (range 1 to 18) days. Mean length of stay for the first bleed was 21.05 hours in Group A and 22.57 hours in Group B. Mean length of stay for the second bleed for Group B was 37.52 hours. There were no significant differences for length of stay or day of presentation for the groups. A summary of timing for each second bleed is presented in Figure 1.

Table 3 compares management of the first bleeding episode between the two groups. Ninety percent of Group A patients were taken to the OR, while only 42.86% of Group B patients were taken to the OR during their first admission, which was a significant difference (*P* < 0.005). Patients not taken to the OR for their first bleed had an odds ratio of 5.78 (95% CI 2.81–11.90) of having a second bleed. For Group B, 90.48% were taken to the OR for their second bleed. Thirty-five patients (38.46%) in Group A and 8 patients (38.1%) in Group B presented with active bleeding to the ER, and the remaining had a history of bleeding or a tonsillar fossa clot. Two patients (2.2%) in Group A and 1 patient (4.8%) in Group B had a history of a primary posttonsillectomy bleed, not a significant difference. Two patients in Group B bled a third time. One was a child with OSA, asthma, and reflux, and one was an adult with suspected tonsil cancer.

We examined the contribution of age (adult versus child), whether the patient was taken to the OR for the first post-op bleed, cardiac history (positive or negative), and indication for tonsillectomy using regression analysis. We found that adults who did not go to the OR for their first post-op bleed, regardless of their cardiac history, and who had an indication for surgery of recurrent infection and suspicion of cancer or both had the highest chance of having a second bleeding episode. Children who were taken to the OR for their first post-op bleed, regardless of their cardiac status or indication for surgery, were least likely to bleed a second time.

## 4. Discussion

Posttonsillectomy hemorrhage can be potentially life threatening. Previous research has explored factors that may increase the risk of developing PTH, but our study investigates only recurrent PTH. Ikoma et al. found adult men were more likely to have PTH [4], but, for recurrent bleeds after a PTH, Liu et al. found that risk factors were female gender and an age of greater than 12 years [6]. Our study group had a higher ratio of males to females, which matches the data found by Ikoma et al. [4]. Likelihood of recurrent bleeding was increased with age, which matches the results previously reported in the literature [4]. We found that the race distribution was more diverse for single-bleed patients, while multiple-bleed patients tend to be mostly Caucasian; however, the difference was not statistically significant.

Data on recurrent PTH has been scarce, but a study by Bhattacharyya and Kepnes reported that 4.8% of adults developed PTH with recurrent PTH in 1.24% [5]. In this study 2.2% of their patients underwent a surgical procedure to control a posttonsillectomy bleed during the first bleed after an initial PTH, while 0.7% underwent a surgical control of the bleed during the second bleed after an initial PTH [5]. Our recurrent bleed rate was slightly lower, at 18.75% of all posttonsillectomy bleeds studied (Bhattacharyya and Kepnes found about 25% of their group rebled) [5]. Our patients were more likely to undergo operative intervention for their bleed.

When taking all indications for tonsillectomy into consideration, the mean number of days after operation that bleeding occurred for recurrent bleeders was 6.1 days for the first bleeding episode and 10 days for the second. Kontorinis and Schwab found about 2.7 days between first and second bleed, although their group appears to have included primary bleeds [7]. This data differs from previous findings by Bhattacharyya and Kepnes, which showed that the most common day for a first bleed is Day 5 and the most common day for a second bleed is Day 6 [5]. Their report may form the basis for a management strategy that includes in-hospital monitoring for 24 hours after a posttonsillectomy hemorrhage; our results dispute this as a reasonable plan in all cases.

We did not find coagulopathies among the patients who bled again. Kontorinis and Schwab reported on coagulation studies in 22 patients with recurrent posttonsillectomy bleeding and had similar findings [7]. In small studies, a rare bleeding disorder may not occur, so whether practitioners should order coagulation studies in patients with one or more posttonsillectomy bleeds is not within the scope of a small study.

Reports in the literature show no significant difference in frequency of PTH between coblation tonsillectomy and diathermy tonsillectomy [8]. We did not find that tonsillectomy method, coblation or cautery, was associated with recurrent PTH but the number of patients who underwent coblation tonsillectomy was small.

Patients who bled recurrently after an initial PTH were less likely to undergo control of their bleeding in the OR during their first admission, which was a statistically significant difference between the groups. Taking a posttonsillectomy bleed patient to the OR at our institution is more likely if the patient is bleeding actively at admission, has a clot in the tonsil fossa, or has bled recurrently. Conversely, patients with no bleeding and no abnormality seen in the tonsil fossae were observed only. None of our patients required complex surgery to control bleeding, compared to Kontorinis and Schwab's group, where 23% required an external neck approach or tracheotomy [7].

Can patients be discharged earlier after operative control of these bleeds? Subsequent bleeds are unlikely—only 10% of those undergoing surgery for a PTH bled again, versus 55% of nonoperative patients—and, for those who bled within the first week, none of these recurrent bleeds occurred within 24 hours of admission. Are patients who bleed more than 7 days post-op different from those who bleed earlier? This is a question for further study.

A further question is whether more patients should be treated operatively when they first present with a posttonsillectomy bleeding, but this answer is beyond the scope of this study.

A retrospective study frequently has limitations to the quality of data, prompting additional studies. For our study, a limited number of patients, especially in Group B, made assessment of the data's significance more difficult. By expanding the size of the study group, future studies will be able to add to the available literature and the understanding of risk factors and time to recurring bleeds for posttonsillectomy patients.

## 5. Conclusion

Our study found that patients with recurrent posttonsillectomy hemorrhages have on average 4 days between bleeding episodes, longer than previously reported in the literature. If patients bleed later than 7 days post-op they are more likely to bleed again within a day, so they may benefit from 24-hour monitoring. Our most important finding is that patients who do not have operative control of their bleeding are more likely to bleed again. Management of posttonsillectomy bleeding does not need to include a 24-hour period of observation solely to monitor for more bleeding in patients who bleed prior to 7 days post-op, especially if the patient is managed operatively.

## Figures and Tables

**Table 1:** Comparison of demographic data.

	Mean age (years)	Mean BMI	Males (%); females (%)	Children (%); adults (%)	Ages 1–5 Yr (%)	Ages 6–15 Yr (%)	Ages 16+ Yr (%)	Race (%)
Group A	16.7 (SD = 11.44)	23.7 (SD = 7.21)	53.3; 46.7	62.2; 37.8	18.0	34.4	46.7	Caucasian: 68.9 African American: 12.2 Hispanic: 8.9 Asian: 3.3 Others: 6.7
Group B	22.3 (SD = 17.70)	26.9 (SD = 7.09)	61.9; 38.1	47.6; 52.4	14.3	28.6	57.1	Caucasian: 85.7 African American: 4.8 Hispanic: 0 Asian: 4.8

Group A: single PTH patients; Group B: multiple PTH patients; there were no significant differences between the groups for these parameters (*P* < 0.05).

**Table 2:** Timeframe data for Group B (multiple PTH) patients, based on indication for tonsillectomy.

	*N* (%)	Days post-op 1st bleed	Mean LOS 1st bleed (hours)	Days post-op 2nd bleed	Mean days between 1st and 2nd bleed
Recurrent tonsillitis	10 (47.6)	5.8	19.6	8.9	3.1
Obstructive sleep apnea (OSA)	6 (28.6)	8.0	21.7	11.3	3.3
Malignancy	3 (14.3)	3.3	37.7	12.7	9.4
OSA + recurrent tonsillitis	2 (9.5)	6.0	17.5	7.5	1.5

LOS: length of stay.

**Table 3:** Comparison of management of PTH.

	Group A (%) *N* = 91	Group B (first PTH) (%) *N* = 21
OR, unilateral cautery	34.4	13.6
OR, bilateral cautery	55.6	23.8
Silver nitrate cautery	3.3	4.8
Observation (24 hr or more)	3.3	19.0
No treatment	3.4	38.8
